# Industrial Dentistry*Read before the Cincinnati Dental Society, January 23, 1920.

**Published:** 1921-01

**Authors:** Carey P. M’Cord

**Affiliations:** University of Cincinnati


### INDUSTRIAL DENTISTRY *

BY CAREY P. M'CORD, UNIVERSITY OF CINCINNATI

Instead of confining ourselves closely to the topic of "Industrial Dentistry," it will be a matter of greater profit to us to consider the general problem of the health of industrial workers, and measures necessary for the health conservation of these workers, of which "Industrial Dentistry" is an integral, but largely undeveloped, part.

### THE SCARCITY OF INDUSTRIAL WORKERS

This country is essentially an industrial country. Forty millions of our people are engaged in industrial activities. Industry has doubled itself in this country during the last fifteen years. Further growth is still needed and desirable, but is hampered through the lack of industrial workers. A generation ago and less there existed an abundance of industrial labor. For every job there was usually more than one applicant. Managements even in selecting their lowest type of workers usually were able to exercise choice in the matter.

Such an abundance of labor no longer obtains. Although our population has increased, industry has increased much more rapidly. Now instead of there being two men for every job there is not even one. During the past decade industry has sought to adequately man its plants through a diversity of means. One means was through the development of man replacing machinery. Such a measure necessarily has limitations beyond which it is not possible to substitute machines for men.

In the search for labor, inducements were offered to hundreds of thousands of foreigners to come to this country to engage in industrial work. More recently overtures have been made seeking to obtain the services of women in industry in large numbers.

* Read before the Cincinnati Dental Society, January 23, 1920.

No one of these measures, nor all together, have been sufficient to stop the increasing shortage of labor.

At the present time we are in another phase of activities on the part of industrial management to adequately fill their plants. This takes the form of competition between plants for the services of labor. This competition assumes a variety of forms in diverse plants and localities. Its fundamental is to be found in making the working conditions, or some aspect of the general working environment, so attractive that workers will spontaneously come to this plant to work. In certain plants this competition takes the form of high wages, in others of bonuses, in others profit-sharing systems, in others shorter hours, etc. At best, such measures are to be regarded as ''patent medicine'' treatment, as they do not offer any hopeful outlook for the solution of any industrial labor problems.

### HUMAN CONSERVATION

In the midst of these competitive measures there has developed, in recent years, a fuller appreciation of the genuine values for all concerned that are to be found in honest, straightforward measures for the conservation of the health of the worker. The need of such measures and the values that are to be found therein, are becoming recognized by the workers themselves, by labor organizations and by industrial managements. It also is recognized by public health workers everywhere that the organization and conduct of industry are such as to offer a splendid vehicle for the application of preventive medicine in all of its aspects.

Every industrial plant contains conditions under which work is performed that are neither conducive to full production for the amount of energy expended by the worker, nor to the health, well-being and long continuation of work on the part of the worker himself. A full knowledge of these deleterious conditions and measures for their eradication have not obtained in the minds either of the workers or the management. Although health hazards have existed in a large number of ways, such as fatigue, overcrowding, faulty ventilation, overheating, poor ventilation and faulty posture, the doctor rendering service to plants has largely concerned himself with the treatment and care of the sick and injured who found their way to the plant's medical office.

Although ninety-eight per cent., or thereabouts, of all industrial workers have mouth conditions that are detrimental to their working efficiency, neither industrial management nor the dental profession has been alert to this need, and to the benefits accruing to all from the application of simple and well-known measures.

Very slowly professional groups and industrial managements are coming to see that their plant needs the services of a human maintenance department, a human conservation department, a human repair department. Such a group, in order to obtain the best results, should be comprised of some or all of the following specialized workers: a trained industrial physician, an industrial dentist, industrial nurses, safety engineers, personnel relations manager, etc.

THE FUNCTION OF THE HUMAN CONSERVATION DEPARTMENT

It is difficult for doctors, for dentists and similar groups to get the viewpoint of industrial managements with reference to their attitude towards their workers. Industrial physicians and dentists, to be of greatest value in industry, must come to realize that they can best serve both the worker and the management of industry by compromising some of the theoretical aspects of medicine and dentistry, and meeting more than half way the conditions that attend industrial production. Industrial physicians and dentists must realize that production is the chief need of industry; that in the shoe factory the chief end to be attained is the manufacture of shoes; if pumps are being made, the chief aim is the production of pumps. In any such plant the health of the workers is significant and important only in that it is a factor in production, and in the advancement and economic betterment of the workers.

To a great extent production is dependent upon the health of the workers and the conditions under which they work. In the majority of plants in this district and in this country a careful investigation reveals that there are few measures in operation for disease and accident prevention; that there is a general lack of adequate medical and surgical care; that work is done in overcrowded and unsanitary working places; that in many places low wages still obtain as well as long hours, and that no educational measures exist leading to healthier working and living conditions. As a necessary result production is brought about by unhealthy and inefficient working forces, subject to many accidents and much disease. General dissatisfaction and discontent lead to widespread low morale throughout the plant. Labor turnover and absenteeism necessarily will be high, and expensive to the management. Production based on any such human foundation can not but be low in quantity and low in quality.

It is the function of this human conservation department to work within this field of bad conditions indicated above; to change this flimsy human foundation of production to a stable and enduring one, which can be accomplished through the institution of disease and accident prevention measures, plant sanitation, educational measures in personal hygiene, the proper selection and placement of men on jobs, the best of medical and surgical care for the sick and injured, and through widespread educational measures in topics pertinent to the health and well-being of the workers.

Such a human conservation department will lead to healthy and energetic working forces—men and women giving full time to their jobs with little time lost through accident and disease, ultimately leading to maximum production by experienced, intelligent, healthy and loyal employes.

In short, the functions of the human conservation departments are:

*First,* to hire physically and mentally fit men and women.

*Second,* to see that they are placed on jobs for which they are physically, mentally and temperamentally fitted.

*Third,* to protect these workers by maintaining plant conditions free from health hazards.

*Fourth,* to keep these workers continually on the job by maintaining their health and efficiency.

### THE INDUSTRIAL DENTIST

In the activities of this group the services of the industrial dentist are very badly needed. The most common of all health hazards in industry are those growing out of faulty mouth conditions. More than ninety per cent. of all workers present oral defects, obviously handicapping them in their work.

It is at once to be recognized that industrial dentistry in its application is not unlike any other form of dentistry. The greatest difference between industrial dentistry and the dentistry applied in the downtown office is to be found in the viewpoint of the dentist. The viewpoint of the industrial dentist is apparent in the following definition of ''industrial dentistry'':

''Industrial dentistry'' is the application of the theory and practice of dentistry to industrial workers in order that they and their employers may enjoy the full benefits of continuous health and work.

The advent of dentistry to industry, however, is so recent that no general agreement exists as to what work should be carried out in the plant by the dentist, or the conditions under which this work should be performed.

In 1914, only about twenty industrial concerns in this entire country utilized the services of dentists in their plants. In 1919, fifty-five plants were known to maintain industrial dental clinics. At that time and thereafter the number was growing so rapidly that no records are available at the present time as to the number of plants employing such services or the types of service rendered therein. It is to be pointed out that at the outset of such activities many errors were made. There was no background to serve as a guide, and this period of pioneer work was necessary in order to form a background for guidance. In earlier days various systems were in effect for the application of industrial dentistry.

*First,* it was found that certain dentists were employed on a full-time or part-time salary; that all equipment and all supplies were furnished by the company, and all service to employes was without charge; the dentists being paid for the time occupied in carrying out dental work. Under such arrangements it was found that the following three classes of work was done:

*A.* Examinations and prophylaxis; all types of fillings, extractions, crown, bridge and plate work—all free to the employe.

*B.* Examination and prophylaxis only, with the necessary dental work of other nature referred to outside dentists under the supervision of the industrial dentist.

C. Educational work and examination only, all work being done by dentists on the outside.

*Second,* dentists employed by industries at a small salary with full equipment supplied and maintained by the company. The dental work was carried out at the expense of the employe, but on company time. Here again a wide variety of arrangements were found to exist.

*Third,* arrangements made by the industry with some nearby outside dentist who supplied dental service of all kinds at reduced rates to the company or to the employe.

At the present time it is practically agreed that the function of the industrial dentist should be limited to the following things:

*First,* educational work in oral hygiene.

*Second,* examination and prophylaxis.

*Third,* emergency filling and emergency work of all kinds.

*Fourth,* participation with the doctor in the physical examination of all employes.

*Fifth,* the supervision of dental work of employes which is carried on by dentists outside the plant.

THE VALUES OF HUMAN CONSERVATION

Each year through ill health or injury every worker loses on the average of eight days. When this figure of eight days is extended to include forty million workers, it means that more than 875,000 years of work is lost each year. No less than seventy-five per cent. of this loss is avoidable, is unnecessary, is preventable. Already in many places the incidence of accidents has been cut down eighty per cent. through the careful application of safety measures. Just so ill health, resulting in lost time, loss of money, and disruption of production, can also be largely eliminated. Widespread ill health results in high labor turnover and high absenteeism. In large American cities the labor turnover averages two hundred per cent. per year. This means that in any plant employing one thousand workers, it is necessary to hire, fire and replace, for all causes, two thousand workers in order to continually maintain a force of one thousand persons. The cost of hiring and firing and training workers is tremendous. For each turnover the cost is not less than sixty dollars. If this figure be applied to two hundred percent. of labor turnover in the one-thousand-man plant, it represents an item of $120,000.

The right type of work on the part of a human conservation department will throw around the workers such conditions in certain industries as will reduce labor turnover to approximately fifty per cent., or one-fourth of that which obtained before the institution of a human maintenance department. This item alone represents a saving of $90,000, which far exceeds the total expense for an entire human conservation department.

It is estimated that if what we know at the present time about health conservation were widely applied, we might expect to add fifteen years of life to the average life of every person. The worker today is not so shortsighted as to overlook the advantages of such measures made in his interest, for any such measures to be profitable to the manufacturer must be applied to the worker and primarily benefit him. Although such things do primarily benefit the worker, ultimately they lead to splendid returns to the manufacturer himself.

---

